# Cyclic Dimers of 4-n-Propyloxybenzoic Acid with Hydrogen Bonds in the Gaseous State

**DOI:** 10.3390/ijms232315079

**Published:** 2022-12-01

**Authors:** Nina I. Giricheva, Ksenia E. Bubnova, Alexander V. Krasnov, Georgiy V. Girichev

**Affiliations:** 1 Nanomaterial Research Institute, Ivanovo State University, 153025 Ivanovo, Russia; 2 Department of Physics, Ivanovo State University of Chemistry and Technology, 153000 Ivanovo, Russia

**Keywords:** 4-n-propyloxybenzoic acid, hydrogen bond, dimer, molecular structure, gas electron diffraction, DFT, mass spectrometry, intermolecular interaction

## Abstract

A comprehensive study of saturated vapors of 4-n-propyloxybenzoic acid (POBA) by gas electron diffraction (GED) and mass spectrometric (MS) methods supplemented by quantum chemical (QC) calculations was carried out for the first time. An attempt was made to detect dimeric forms of the acid in the gaseous state. It has been established that at the temperature of GED experiment, vapor over a solid sample contains up to 20 mol.% of cyclic dimers with two O-H...O hydrogen bonds. The main geometrical parameters of gaseous monomers and dimers of POBA are obtained. The distance r(O…O) = 2.574(12) Å in the cyclic fragment of the gaseous dimer is close to that in the crystal structure (2.611 Å). In the mass spectrum of the POBA recorded the ions of low intensity with a mass exceeding the molecular mass of the monomer were detected. The presence of ions, whose elemental composition corresponds to the dissociative ionization of the dimer, confirms the results of the GED experiment on the presence of POBA dimers in the gas state. The results of GED studies of acetic acid, benzoic acid, and POBA were compared. It is shown that the COOH fragment saves its geometric structure in monomers, as well as the COOH...HOOC fragment with two hydrogen bonds in dimers of different acids. The intermolecular interaction energy in considered acid dimers was estimated using QC calculations (B97D/6-311++G **). The significant value of last (>84 kJ/mol) is the reason for the noticeable presence of dimers in the gas phase.

## 1. Introduction

It is well known that aromatic carboxylic acids are dimerized in crystalline and liquid crystalline phases [1,2,3,4,5,6]. Due to this feature, even benzoic acids with short substituents can exhibit liquid crystal properties [5].

During heating, substituted benzoic acid passes into a liquid-crystalline state, and then into an isotropic state with a gradual destruction of intermolecular hydrogen bonds, and according to IR spectroscopy, the number of dimers in the isotropic state becomes small [1].

For aromatic carboxylic acids the method of IR spectroscopy is a reliable tool for detecting dimeric forms of acids, since IR spectra contain characteristic absorption bands in the range of 2500–2700 cm^−1^ [3,4,6,7,8] and a band related to the frequency of the C=O stretching vibration (1680–1700 cm^−1^), which is shifted to the low-frequency region of the IR spectrum compared with the frequency ν(C=O)_st_ for the monomeric form, which is usually localized at 1740 cm^−1^ [1,3,4]. Most of the IR spectra of aromatic carboxylic acids refer to samples in the crystalline state, or to a solution of acids in non-polar solvents, in which intermolecular hydrogen bonds are preserved.

An interesting question is the possibility of the existence of dimers of benzoic acids in vapor. The experimental methods that can be used divulge the answer are IR spectroscopy of the gas phase, mass spectrometry and gas electron diffraction. In this case, the temperature of the experiment and the total vapor pressure are very important, since the dimerization constant K_dim_ strongly depends on these parameters, in addition, the method of registration of dimeric forms is also important.

It is reported in [9,10,11,12,13] that substituted benzoic acids can be in the dimeric form in the gas phase. This conclusion is mainly based on the interpretation of the vibrational spectra of benzoic acid (BA) and its substituted ones obtained under special conditions.

For example, in [10], the IR absorption spectra of the jet-cooled BA monomer and dimer, recorded throughout the 500–1900 cm^−1^ range via ion dip spectroscopy, was obtained. In [13], the IR spectra of the BA dimer have been recorded under jet-cooled conditions using the double resonance method of fluorescence-dip IR spectroscopy. The authors [13] note that “the spectra are assuredly due exclusively to dimers in the ground-state zero-point level at a rotational temperature of 3–5 K”. Even under these conditions, the dimers have remarkably broad spectra, extending from 2600 to almost 3150 cm^−1^.

In [11,12], the conclusion about the existence of dimers was made based on the infrared spectra of benzoic acid and deuterobenzoic acid embedded in Ar matrices; the compounds were evaporated from a Knudsen effusion cell at 46 °C. The authors note that both a raise of benzoic acid concentration and a matrix annealing lead to the formation of H-bonded benzoic acid dimers.

At the same time, in NIST Chemistry WebBook, the IR spectra of gaseous BA and its substituted ones did not reveal bands characteristic of dimers. However, it should be noted that the IR spectra of the gas refer to the temperature of the experiment at which complete thermal dissociation of dimeric forms is possible, for example, for unsubstituted BA, the gas temperature is 160 °C.

Therefore, for the study of monomer-dimer equilibrium, the method of GED may be more effective, which requires lower experimental temperatures than IR spectroscopy of gaseous compounds, and when registering a diffraction pattern, there is no destructive effect on the molecular forms present in the vapor under study, such as this is the case in electron ionization mass spectrometry.

Such electron diffraction studies of BA were performed in two laboratories at different vapor temperatures (T = 288 K [14] and T = 405 K [15]). In both works, the interpretation of the diffraction pattern was made on the assumption of a monomer composition of the vapor. The possibility of the presence of dimeric forms in the vapor was not tested.

Thus, in the literature, there is no consensus about the possibility of maintaining hydrogen bonds (HBs) in gaseous aromatic carboxylic acids.

At the same time, for acetic acid (AA), the presence of cyclic dimers with two hydrogen bonds in vapor was proved by the electron diffraction method. A unique example of such a study is the work [16], in which a two-temperature electron diffraction/mass-spectrometric (GED/MS) experiment was performed. It is shown that at T = 296 K the gas phase contains dimeric forms in the amount of 31(2) mol%, while at T = 457 K, the number of dimers is negligibly small. The validity of this conclusion is confirmed by the distinct difference between the two radial distribution functions f(r) associated with the different composition of the gas phase, i.e., with different monomer-dimer ratio and its dependence on the temperature. At the same time, the mass spectra of acetic acid obtained at two temperatures in [16] did not contain ions with a mass greater than the molecular mass of the monomer.

The purpose of this work is a comprehensive study of vapors of 4-n-propyloxybenzoic acid (POBA) by electron diffraction, mass spectrometric and quantum chemical methods and an attempt to detect dimeric forms of the acid in the gaseous state. The object of study is the first representative in the series of 4-n-alkyloxybenzoic acids (*n* ≥ 3), which exhibits liquid crystal properties, despite the presence of a short substituent –OC_n_H_2n + 1_.

An encouraging example of a gaseous acetic acid dimer [16], on the one hand, inspires hope for a successful solution of the problem of establishing the monomer–dimer composition of POBA vapors, since, according to our estimates, the intermolecular interaction energy (E_IMI_) in the POBA dimer is comparable to that for the acetic acid dimer. On the other hand, there are circumstances that complicate this task, since the POBA dimer is not an easy object to study by the GED method, since dimer contains internuclear distances exceeding 20 Å.

## 2. Results and Discussions

### 2.1. Peculiarity of GED Data Structural Analysis of POBA Vapor

The temperature 87(15) °C of GED experiment was lower than the temperature of “crystal-liquid crystal” transition (T_Cr→LC_ = 140.5 °C), i.e., POBA sublimation took place.

The theoretical function of molecular scattering sM(s) contained two terms:

sM(s) = α∙sM(s)_mon_ + (1−α)∙sM(s)_dim_ (1)

where sM(s)_mon_ is the theoretical function for monomer, sM(s)_dim_ is the theoretical function for dimer, α is the coefficient that determines the contribution of the monomeric form to the function sM(s), which can take the value 0 < α < 1.

The coefficient α is related to the mole fraction of the monomer by the following relationship χ_mon_ = α/[α + (1−α)/3.54], which takes into account the different scattering power of monomeric and dimeric forms. The scattering power of molecular species present in the vapor were estimated by the formula: ΣZ_i_Z_j_ × k_ij_/r_ij,_ where Z_i_ are charges of atom nuclei; r_ij_ is the distance between i and j atoms; k_ij_ is the multiplicity of the (i–j) distance. The ratio of scattering power of dimer and monomer calculated in this way is 3.54/1.

**Figure 1:**
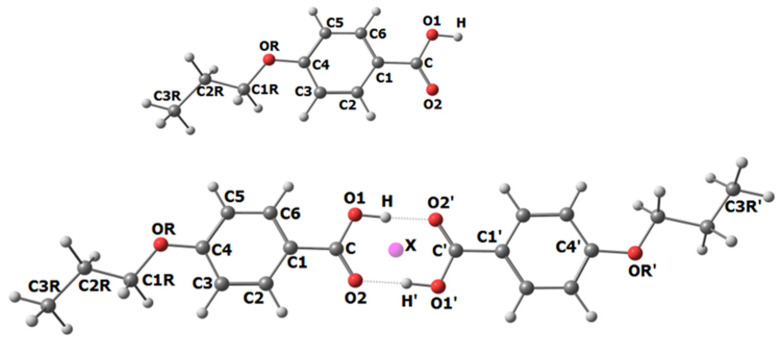
Geometric models of the POBA monomer and dimer with atomic numbering.

The geometric models of the monomer and dimer with the numbering of atoms are shown in Figure 1. To describe their geometry, 21 independent parameters were used: five internuclear distances (C-O1, O1-H, C1-C, C2-H, C…X), eleven bond angles (C-O1-H, O2=C-O1, C1-C-O1, C-C1-C2, C-C1-C6, C4-OR-C1R, C1-C2-H, OR-C1R-H, H-C1R-H, O2=C-X, O1-C-X), and five torsion angles (C1R-OR-C4-C5, C2R-C1R-OR-C4, C3R-C2R-C1R-OR, H-C1R-OR-C4, H-C3R-C2R-C1R). The non-equivalence of C-C and C-H bonds in benzene and propyl fragments was taken into account.

In the least squares analysis of electron diffraction data (modified KCED program [17]), the parameters obtained from the results of quantum chemical calculations (B97D/6-311++G **) were taken as the starting values of internuclear distances, bond angles and torsion angles. VibModule program [18] was applied to calculate the vibrational corrections Δ*r* = *r_h_*_1_ − *r_a_* and the starting values of root-mean-square vibration amplitudes of monomer and dimer POBA at the temperature of the GED experiment using the harmonic approximation and taking into account the non-linear interrelation between internal and Cartesian vibrational coordinates. The quantum chemically calculated difference between parameters of the same type in the monomer and dimer was retained. In the least squares analysis, both 10 geometrical parameters (Table 1) along with 14 groups of vibration amplitudes of the monomer and dimer were varied simultaneously.

### 2.2. Conformers of the Monomeric and Dimeric Forms of POBA. Possibilities of Gas Electron Diffraction Method

In [2,19], we considered conformers of the POBA monomer, which differ both in the spatial structure of the OC_3_H_7_ substituents and in the structure of the COOH acid group. The latter can have a *cis-*(H-O1-C=O2 torsion angle is 0°) or *trans-*(H-O1-C=O2 torsion angle is 180°) conformation (Figure S1). The most energetically favorable (by ≈25 kJ/mol, B97D/6-311++G **) is the *cis*-configuration which can form cyclic dimers.

In addition, the POBA monomer has some conformers that differ in the structure of the –OC3H7 substitute. The most stable conformers, the energy difference of which does not exceed 6.7 kJ/mol, are shown on Figure S2.

The calculation results showed that, regardless of the spatial structure of the substituents, the intermolecular interaction energy, and the geometry of the –OPh-COOH···COOH-PhO- backbone in the POBA dimer remain the same.

For GED analyze the model of POBA monomer with a flat *trans*-structure of the substituent carbon backbone was chosen (Figure 1). This conformer is energetically more favorable.

The set of independent geometric parameters includes torsion angles C1R-OR-C4-C5, C2R-C1R-OR-C4, C3R-C2R-C1R-OR, H-C3R-C2R-C1R (see Section 2.1). Their variation gives effective values, which in their physical meaning correspond to thermally averaged values of torsion angles for all monomeric conformers (Figure S2) present in saturated vapor at the temperature of the GED experiment. The variation of the four indicated torsion angles does not lead to a decrease in the disagreement factor R_f_ but indicates a large error in their determination and shows the impossibility to determine substituent conformations in the monomeric and dimeric forms of the acid by the GED method.

It should be noted that the GED method is quite sensitive to determination of structural parameters of molecules with a set of internuclear distances in the range from 1 to 15 Å. If molecular forms have terms with internuclear distances r > 10 Å, then to them determination, it is necessary to decrease the scanning step Δs of the electron scattering intensity sM(s).

In [20], we considered the question of the relationship between the scanning step Δs and the maximum value of the internuclear distance r, as well as the reliability of determining terms corresponding to large r values. It was shown that even when the scanning step Δs is reduced to 0.1 Å^−1^, instead of the traditionally used 0.2 Å^−1^ or 0.25 Å^−1^, the sensitivity of the GED method is very low for terms with internuclear distances r > 14 Å.

In the case of POBA, the monomeric form has an effective length of about 12 Å, the dimer is 24 Å, and the -Ph-COOH…HOOC-Ph- dimer synthon is 12 Å. The range of distances from 1 to 12 Å also includes terms which belong to different monomers in the dimer (for example C…C3R’, C1…OR’, OR…C4′ (Figure 1)).

Thus, the set of terms determined by the GED method includes the terms of the monomer, the terms of monomer fragments in the dimer, and the terms describing the central part of the dimer. However, it is not possible to reliably determine the relative positions of the two –OC_3_H_7_ substituents in the dimer.

These limitations in studying the composition and geometry of the POBA vapor components affected the found set of geometric parameters of monomers and dimers (Table 1).

**Figure 2:**
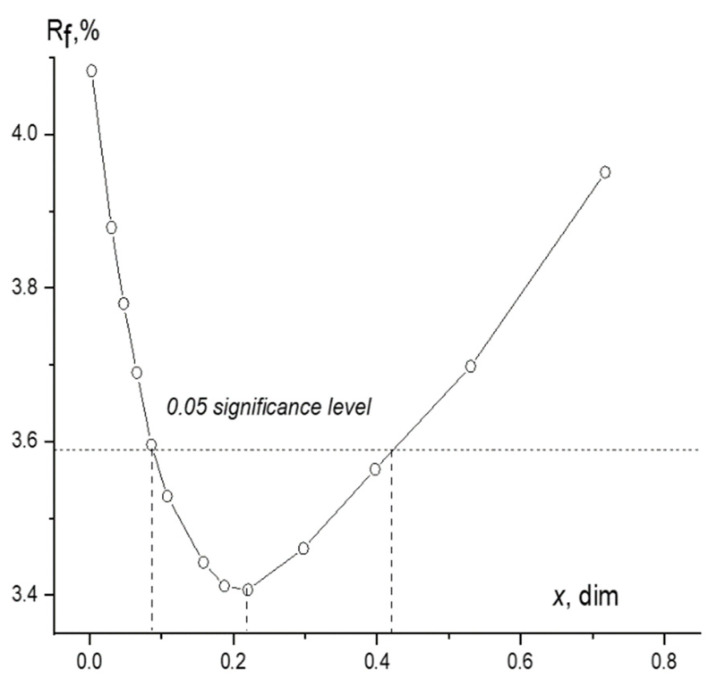
Dependence of the value of R_f_ on the mole fraction of the dimer χ_dim_ in the vapor of POBA The horizontal dashed line corresponds to the Hamilton’s test [21] for uncertainties at significance level 0.05.

Note that two options for determining the vapor composition were used: the grid method, in which the coefficient α in equation (1) changed with a certain step when refining all other independent parameters (Figure 2), and the simultaneous variation of the structural parameters and the coefficient α associated with the concentration monomers and dimers in the framework of the least-squares analysis.

Both options lead to results consistent within the error (simultaneous variation of χ_dim_ = 0.20(10), R_f_= 3.405%; grid method χ_dim_ = 0.22, R_f_ = 3.407%, Figure 2).

**Figure 3:**
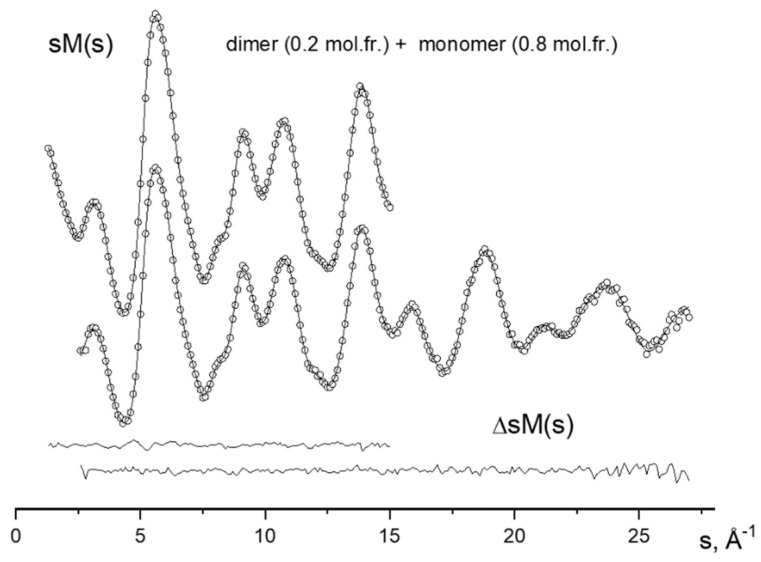
Experimental (points) and theoretical (lines) curves of the molecular scattering intensity sM(s) and their difference.

**Figure 4:**
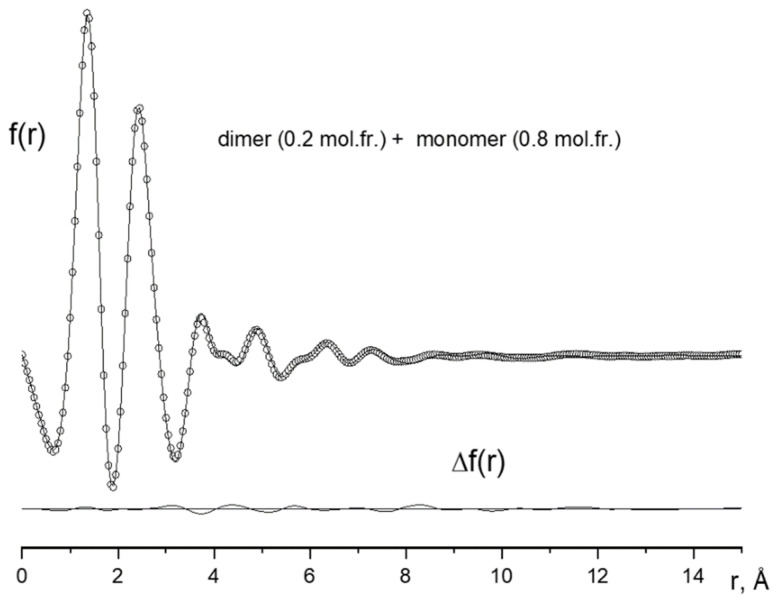
Experimental (points) and theoretical (lines) curves of the radial distribution f(r) and their difference ∆f (r).

The experimental functions of the molecular scattering intensity sM(s) and the radial distribution f(r) together with theoretical analogs corresponding to the optimized values of the structural parameters and the optimal ratio between the monomers and dimers of POBA vapor are shown in Figure 3 and Figure 4, respectively.

### 2.3. Mass Spectrometric Confirmation of the Existence of Dimeric Forms in a Vapor over POBA

To date, we are not aware of mass spectrometric data that would indicate the presence of dimeric forms of any aromatic carboxylic acids in the gas phase. The NIST database lists the mass spectrum of POBA in the range *m*/*z* = 0–180, which does not cover the mass range of ions related to the dimeric forms of the acid (Figure S3).

From the example with acetic acid, it is known that its dimers decompose upon electron impact and are not detected in the mass spectrum [16].

We have carried out a mass-spectrometric study of POBA vapors with a thorough analysis of the high mass range.

In the synchronous GED/MS experiment, the mass spectrum recorded simultaneously with the registration of the diffraction pattern in the extended mass range also did not contain intense ions belonging to dimeric form.

However, the APDM-1 mass spectrometer, which is part of complex GED/MS, has a low sensitivity. Therefore, the mass spectrometric experiment for POBA was performed on an MI-1201 device [22], which has both a higher sensitivity and resolution compared to APDM-1.

**Figure 5:**
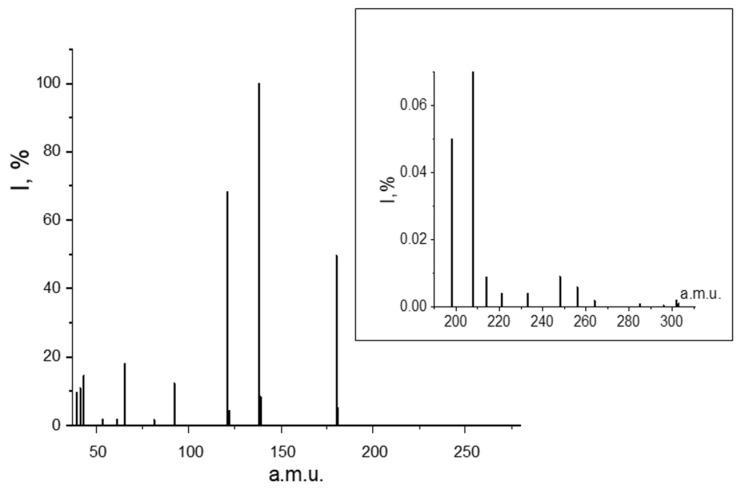
Mass spectrum of POBA recorded on an MI-1201 device.

As well as in the GED/MS experiment, the ions corresponding to the monomeric form of the acid were registered in the range of 0–180 Da. The POBA monomer has a molecular weight of 180 a.m.u. In addition to them, when scanning the range of 180–400 a.m.u., heavy ions of low intensity were detected (Figure 5).

The elimination of certain fragments during the ionization of molecules by electrons, known in the mass spectrometry of organic compounds [23], was used to identify elemental composition of ions.

It was supposed the elimination of the phenyl fragment -C_6_H_4_- and elimination of groups –CH_2_–, –CH_2_-CH_2_–, –CH_2_-CH_2_-O–, –CH_2_-CH_2_-CH_2_-O– from substituents –O-C_3_H_7_.

The possible elemental composition of monomer ions as well as ions with masses of more than 180 a.m.u. found in the mass spectrum of the acid are shown in Table 2. The presence of such ions corresponding to the dissociative ionization of the dimer confirms the results of the GED experiment on the presence of POBA dimers in the gas phase.

### 2.4. Comparison of the Results of GED Studies of Three Carboxylic Acids: AA—Acetic Acid, BA—Benzoic Acid, and POBA—4-n-Propyloxybenzoic Acid

Two-temperature GED studies of AA vapors were carried out in 1944, 1971, and 2020 at three GED laboratories [16,25,26].

The main conclusion of these studies is the statement that AA vapor at temperatures close to room temperature contains a significant amount of dimeric form with two HBs, and at temperatures 120–150° higher, the amount of dimers in the vapor is negligibly small.

For unsubstituted benzoic acid BA, studies were also performed at two temperatures (288 K [14] and 405 K [15]). However, they were performed in different GED laboratories on devices with different inlet systems. Moreover, the construction of new evaporator for low pressure gas electron diffraction was used in [14]. As was noted in the introduction, the interpretation of the GED data [14,15] obtained at different temperatures was performed on the assumption that only monomeric forms of BA are present in the vapor.

Similar to the results for AA, one would expect the presence of dimeric forms of BA at low temperatures [14]. However, the very small value of the structural wR factor of 2.52% indicates a good agreement between the theoretical monomer model of BA vapor and the experimental data.

Using the DFT/B97D/6-311++G ** method, we calculated the geometrical and vibrational parameters (T = 298 K) and theoretical functions sM(s) and f® of the BA monomer and dimer (Figures S4 and S5).

It can be seen the theoretical functions sM(s)_dim_ and sM(s)_mon_ have common visual features, which are also observed on the experimental function sM(s) [14]. At the same time, the difference curve ΔsM(s) = sM(s)_d–m_ − sM(s)_mon_ is significantly different from zero (Figure S4).

Significant differences are seen between the theoretical functions f(r)_mon_ and f(r)_dim_ (Figure S5). Thus, the GED method would make it possible to detect the presence of dimers in the BA vapor.

For further analysis of the results [14,15], we estimated the mole fraction of the dimer in BA vapor under the conditions of the GED experiments (T and P were taken from [14]). Using the DFT/B97D/6-311++G ** method, we calculated the Gibbs free energy of the dimerization process at different temperatures (ΔG_290_ = −19.5 kJ/mol and ΔG_405_ = −0.75 kJ/mol). It turned out that the equilibrium constant k_x_ = x_dim_/x^2^_mon_ of “2·monomer↔dimer” reaction is 6·10^−4^ and 0.018 at T = 290 K and T = 405 K, respectively. Small values of k_x_ can be an explanation for the absence of dimeric forms in BA vapor for experiments [14,15]. The low vapor pressure in the evaporator in the first experiment [14] and the high vapor temperature in the second experiment [15] contributed to the dissociation of dimers into monomers.

Table 3 compares the geometric parameters obtained by GED experiments for the monomeric forms of POBA [this work], BA [14,15], and AA [16], as well as the parameters of the cyclic COOH∙∙∙HOOC fragment in POBA and AA dimers.

Despite the fact that the parameters for the monomers AA, BA, and POBA are different in their physical meaning, they all agree within the experimental error. Agreement is also observed between the parameters of the cyclic fragment in the AA and POBA dimers.

Therefore, it can be concluded that the COOH fragment retains its structure in the monomers of various carboxylic acids, as does the COOH…OOC fragment with two hydrogen bonds in the dimers.

These experimental data are confirmed by the results of quantum chemical calculations on the structure of monomeric and dimeric forms, characteristics of HB, and dimerization energy of the considered carboxylic acids (Table S1).

During the formation of cyclic dimers, as noted in [16,25,26], the C=O and O–H bonds are lengthened, while the C–O bonds are shortened, which can be clearly explained using NBO analysis.

### 2.5. NBO-Analysis and HB Energy

In [27], we analyze the nature of the HB in the POBA dimer and estimate the energy of the HB using the NBO analysis of the electron density distribution.

The geometric parameters of the O-H∙∙∙O=C fragments and the dimerization energy correspond to a strong HB between the carboxylic acid monomers. Note that the distance r(O∙∙∙O) in gaseous dimers (Table 3) is close to that in the crystal structure POBA (2.611 Å [28]).

The main donor-acceptor interactions occur between the lone electron pairs of the LP(O2) atom and the antibonding orbital σ*(O1′-H’), which lead to the formation of a bonding region between O2∙∙∙H’ atoms, to a decrease in the electron density in the region of C=O2 and O1′-H’ bonds, and an increase their length.

The balance between the energy of the donor-acceptor interaction of the orbitals E^(2)^ = 131.0 kJ/mol and the energy of steric repulsion E_steric_ = 79.2 kJ/mol between the electron density of the orbitals LP(O2) and the bonding orbital σ(O1′-H’) is 51.8 kJ/mol. The latter value can be considered as the energy of one HB in the POBA cyclic dimer (Table S2).

On the other hand, the HB energy can be estimated from the intermolecular interaction energy ∆E_IMI_, calculated as the difference between the optimized dimer energy E_dim_ and the energies of the monomers with their geometry in the complex E_mon_(SP). To calculate the latter, the SP (Single Point) variant was used.

∆E_IMI_ = E_dim_ − 2E_mon_(SP)

The interaction energy ∆E_IMI_ is −88.7 kJ/mol, i.e., more than 44 kJ/mol per HB. Close values of this energy were obtained for p-n-propyloxycinnamic and p-n-propylbenzoic acids in [29] (see Note S1).

The significant value of the intermolecular interaction energy in carboxylic acid dimers is the reason for their noticeable presence in the gas phase.

## 3. Materials and Methods

Commercial (Aldrich) 4-n-propyloxybenzoic acid was used without additional purification.

### 3.1. Conditions of a Synchronous GED/MS Experiment

The gas phase electron diffraction patterns and mass spectra were recorded simultaneously using the technique described in ref. [30,31] at two nozzle-to-plate distances (338 and 598 mm). The POBA sample was evaporated at a temperature of 87(15) °C from an effusion cell made of X18H10T stainless steel. The cell nozzle size is 0.5 × 1.6 mm (diameter × length).

The conditions of the GED/MS experiments are shown in Table 4.

For each distance nozzle-to-plate, 6 electron diffraction patterns of the studied substance and 2 diffraction patterns of the ZnO polycrystalline standard were obtained. The latter were taken before and after recording electron diffraction patterns of POBA vapors to control and accurately determine the electron wavelength.

### 3.2. The Separate MS Experiment

MS experiment was carried out on a serial MI-1201 mass spectrometer equipped for thermodynamic studies [22]. In the study of saturated vapors over POBA, a molybdenum effusion cell was used with the ratio “evaporation area/effusion orifice area” equal to 1000. The ionizing voltage in the ion source was 50 V. The temperature of the effusion cell was measured using a tungsten-rhenium thermocouple WRe-5/20.

### 3.3. Computational Details

The QC calculations were carried out using the Gaussian 09 software [32], within the density functional theory (DFT) using B97D [33] functionals with 6-311++G ** basis sets [34]. The use of the functional, which considers dispersion interaction, is important in theoretical studies of the considered objects capable of forming H-bonded complexes. The results of the QC calculations were visualized with the ChemCraft program [35]. The electron density distribution was analyzed using the NBO program [36] from the Gaussian 09 software.

The results of QC calculations were used for a comparative analysis of the geometric parameters, electronic, and vibrational characteristics of the monomeric and dimeric forms of acetic, benzoic, and POBA acids, in the calculation of thermodynamic functions at different temperatures.

For each molecular form, the geometry was optimized without any restrictions, the vibration frequencies and the force field were calculated in the harmonic approximation. The latter were used to calculate the amplitudes of vibrations and corrections to internuclear distances necessary for the structural analysis of GED data. In this case, a modified VibModule program [18] was used (for details of the modification, see note S2), which makes it possible to calculate the vibrational parameters for supermolecules containing intermolecular hydrogen bonds.

## 4. Conclusions

Performed study of the saturated vapor of p-n-propyloxybenzoic acid showed that an efficient method for detecting hydrogen-bonded carboxylic acid dimers is the electron diffraction method.

For recording gas IR spectra, a higher vapor pressure is required than in electron diffraction experiment, hence high temperatures, which lead to the dissociation of dimers. In the method of electron impact mass spectrometry, deep dissociative ionization of dimers is observed, and fragment ions with a mass exceeding the mass of the monomer have a very low intensity compared to monomeric ions, based on which a false conclusion can be made about the composition of the vapor.

One of the advantages of the GED method is the greater scattering power of dimers compared to monomers. This makes it possible to detect dimeric forms even with a significant predominance of monomers in the vapor.

It is shown that the geometrical parameters of the cyclic fragment of a dimer with two hydrogen bonds can be determined by the GED method. However, this method is not sensitive to the determination of conformations of OC_3_H_7_ substituents.

The decisive role in the successful registration of dimeric forms in aromatic carboxylic acid vapors by the GED method is the choice of experimental conditions, which depend on the design of the inlet system used.

The QC modeling is a necessary tool that supplements experimental studies in solving such problems. It is shown that due to the presence of two strong hydrogen bonds, not all cyclic dimers of the considered acids dissociate into monomers during sublimation.

## Figures and Tables

**Table 1:** Theoretical (B97D/6-311++G **) and experimental (GED) geometric parameters of the gaseous monomer and cyclic dimer POBA (R_f_ = 3.405%).

	Monomer		Dimer	
Parameters ^a^	QC r_e_ Structure	GED ^b^ r_h1_ Structure	QC r_e_ Structure	GED r_h1_ Structure
(C-C)_av_ (Ph)	1.404	1.405(4) *p*_1_*^c^*	1.405	1.406(4)
C-O1	1.375	1.368(7) *p*_2_	1.332	1.325(7)
C=O2	1.219	1.212(7) (*p*_2_)	1.242	1.234(7)
O1-H	0.971	0.971	1.010	1.010
C1-C	1.483	1.484(4) (*p*_1_)	1.484	1.484(4)
C1-C6	1.413	1.414(4) (*p*_1_)	1.413	1.414(4)
C-H_av_(Ph)	1.087	1.082(5) *p*_3_	1.087	1.082(5)
C4-O_R_	1.362	1.354(7) (*p*_2_)	1.362	1.355(7)
O_R_-C1_R_	1.443	1.436(7) (*p*_2_)	1.442	1.435(7)
C1_R_-C2_R_	1.524	1.525(4) (*p*_1_)	1.524	1.525(4)
C-Hav (R)	1.101	1.094(5) (*p*_3_)	1.099	1.095(5)
O2=C-O1	121.6	121.8(30) *p*_4_	123.2	123.2(30)
C1-C-O1	112.9	114.4(13) *p*_5_	114.6	113.1(13)
H-O1-C	105.9	105.9	110.1	110.1
C6-C1-C	123.0	122.9(4) *p*_6_	122.1	122.0(4)
H-C6-C1	119.6	124.6(18) *p*_7_	119.4	124.3(18)
C4-O_R_-C1_R_	118.5	117.4(23) *p*_8_	118.5	117.4(23)
H-C1_R_-O_R_	109.3	111.6(80) *p*_9_	109.4	111.6(80)
C2-C1-C	118.3	116.5(25) *p*_10_	119.1	119.0(25)
O1-C-C1-C2	180.0	180.0	179.2	179.2
C5-C4-O_R_-C1_R_	180.0	180.0	178.6	178.7
C3_R_-C2_R_-C1_R_-O_R_	180.0	180.0	179.0	179.0
H-C1_R_-O_R_-C4	−59.2	−59.2	−60.0	−62.9
H-C3_R_-C2_R_-C1_R_	180.0	180.0	180.0	180.0
χ, mol.fr.		0.80(10)		0.20(10)

^a^ distance in Å, angle in degree. ^b^ Uncertainties given in parentheses were taken as: [(2.5σ_LS_)^2^ + σ^2^_scale_]^1/2^, σ_scale_ = 0.002r for bond distances and 3σ_LS_ for bond angles, σ_LS_ is a standard deviation in the least-squares refinement. *^c^ p_i_*—refinable independent parameter; (*p_i_*)—parameter refined in the *i*-th group.

**Table 2:** Ions recorded in the mass spectrum of POBA in this work (MI-1201) and NIST.

Ion Mass a.m.u.	Elemental Composition of Ion ^a^	I, % this Work	I, % NIST
39	C_3_H_3_	10	22
41–43 ^b^	C_3_H_n_	15	21
50–53	C_4_H_n_	2	8
63–65	C_5_H_n_	18	23
81	C5H4OH	2	7
93	C_6_H_4_-OH	12	12
121	HOOC-C_6_H_4_	68	92
138	HOOC-C_6_H_4_-OH	100	100
180	HOOC-C_6_H_4_-OC_3_H_7_	50	25
198 ^c^	–COOH….HOOC–C_6_H_4_-OCH_3_	0.05	
207	H_7_C_3_O–COOH….HOOC–OC_3_H_7_	0.14	
214–212	H_3_CO–COOH….HOOC–C_6_H_4_-CH_3_	0.01	
221–222	H_6_C_3_–COOH….HOOC–C_6_H_4_-CH_2_ -C_6_H_4_-**B**-C_6_H_4_-CH_2_-	0.004 <0.01	
233–232	H_5_C_6_-**A**-C_6_H_4_-OH	<0.01	
248	HO-H_4_C_6_-**A**-C_6_H_4_-OH	0.01	
256–258	H_7_C_3_O–COOH….HOOC–C_6_H_4_-OCH_3_	<0.01	
264	H_4_C_2_-C_6_H_4_-**B**-C_6_H_4_-C_2_H_4_	<0.01	
285	H_7_C_3_O–COOH….HOOC–C_6_H_4_-OC_3_H_7_	<0.01	
296	H_4_C_2_O-C_6_H_4_-**B**-C_6_H_4_-OC_2_H_4_	<0.01	
302–300	H_2_CO-C_6_H_4_–COOH….HOOC–C_6_H_4_-OCH_2_	<0.01	

^a^ **A** –O=C-O-C=O–; **B** –O=C-C=O–. ^b^ The maximum intensity of ions in the C_m_H_n_ groups is given. ^c^ It was noted that the molecular ion of aliphatic carboxylic acids dimer is also not registered in the mass-spectra [24], but the M·COOH ion, which contains the COOH…HOOC fragment, is registered.

**Table 3:** The selected geometric parameters of AA, BA and POBA obtained by GED.

	POBA this Work		AA [16] ^a^		BA [14,15] ^a^	
Parameters	Monomer r_h1_ Structure T = 360 K	Dimer r_h1_ Structure T = 360 K	Monomer r_e_ Structure T = 296 K	Dimer r_e_ Structure T = 296 K	Monomer r_a_ Structure T = 288 K	Monomer r_a_ Structure T = 405 K
(C_Ph_-C_Ph_)_av_	1.405(4)	1.406(4)	-	-	1.393(12)	1.401(2)
C-O	1.368(7)	1.325(7)	1.350(8)	1.315(10)	1.350(10)	1.367(8)
C=O	1.212(7)	1.234(7)	1.207(10)	1.226(12)	1.205(10)	1.225(6)
C1_Ph_-C	1.484(4)	1.484(4)	1.490(12)	1.493(12)	1.493(10)	1.484(7)
C-H_av_(Ph)	1.082(5)	1.095(5)	1.090(17)	1.090(17)	1.103(10)	1.102(8)
O…H		1.565(8)		1.657(23)		
O…O		2.574(12)				
O–C=O	121.8(30)	123.2(30)	120.5(9)	123.0(12)	-	120.3(31)
χ_mon_	0.80(10)		0.69(5)		1.0	1.0

^a^ For a correct comparison in brackets instead of σ_LS_ [14,16] the uncertainties in internuclear distances calculated by the formula σ = [(0.002r)^2^ + (2.5σ_LS_)^2^]^1/2^ is used.

**Table 4:** Conditions of the synchronous GED/MS experiments.

Nozzle-to-Plate Distance, mm	338	598
Number of recorded plates	6	6
Electron beam current, μA	1.35	0.65
Wavelength of electrons, Å	0.03969(3)	0.03981(4)
Temperature of the effusion cell, K	360(15)	360(15)
Average exposure time, s	105	85
Residual gas pressure in diffraction chamber, Torr	1.3 · 10^−6^	1.8 · 10^−6^
s-range/∆s, Å^−1^	2.6–27.0/0.1	1.3–15.0/0.1
Ionization voltage, V	50	50

## Data Availability

Not applicable.

## Supplementary Materials

The following supporting information can be downloaded at https://www.mdpi.com/article/10.3390/ijms232315079/s1.
