# Supplementary material for: Cyclic Dimers of 4-n-Propyloxybenzoic Acid with Hydrogen Bonds in the Gaseous State
Source: Int J Mol Sci. 2022 Dec 1;23(23):15079. doi: 10.3390/ijms232315079 (PMC9737191; doi:10.3390/ijms232315079)
Supplement: Supplementary file 1 [file ijms-23-15079-s001.zip › ijms-2039038-supplementary.pdf]

SUPPLEMENTARY MATERIALS

Cyclic dimers of 4-n-propyloxybenzoic acid with hydrogen bonds in the gaseous state.

Nina I. Giricheva [1,*], Ksenia E. Bubnova [2], Alexander V. Krasnov[2], Georgiy V. Girichev [2]

[1] Nanomaterial Research Institute, Ivanovo State University, Ermak street 39, 153025 Ivanovo, Russia; n.i.giricheva@mail.ru

[2] Department of Physics, Ivanovo State University of Chemistry and Technology, Sheremetevsky Avenue 7, 153000 Ivanovo Russia; 31ksenia94@gmail.com (K.E.B.); kracnov@list.ru (A.V.K.); g.v.girichev@mail.ru (G.V.G)

Content

1. Figure S1. Cis- (torsion angle H-O-C=O is 0°) and trans- (torsion angle H-O-C=O is 180°) conformations of COOH-group ……………………………………………………………..2
2. Figure S2. Conformers differing in the structure of the –OC_3H_7 substituent ………………….2
3. Figure S3. Mass spectrum of p-n-propyloxybenzoic acid (POBA) from the NIST database…..2
4. Figure S4. Theoretical curves of the molecular scattering intensity sM(s) of monomer, dimer of BA acid and their difference ΔsM(s). ……………………………………………... 3
5. Figure S5. Theoretical curves of the radial distribution f(r) of monomer, dimer of BA acid and their difference Δf (r). …………………………………………………………………... 3
6. Table S1. Geometric characteristics of HBs and energy of dimerization of carboxylic acids according to the results of quantum chemical calculations ……………………….. ………. 4
7. Table S2. Energy of donor-acceptor interaction of orbitals $E^{(2)}$ and energy of steric repulsion E_{steric} in POBA acid dimer (in kJ/mol) ……………………………………………........ 4
8. Note S1. The energies of intermolecular interaction in different aromatic carboxylic acids… 5
9. Note S2. Details of VibModule program modification for calculation of the vibrational parameters of supermolecules containing intermolecular hydrogen bonds……………………5

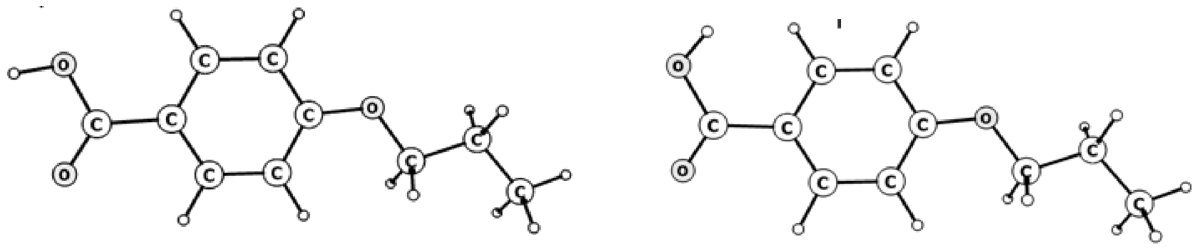

Figure S1. Cis- (torsion angle H-O-C=O is 0°) and trans- (torsion angle H-O-C=O is 180°) conformations of COOH-group

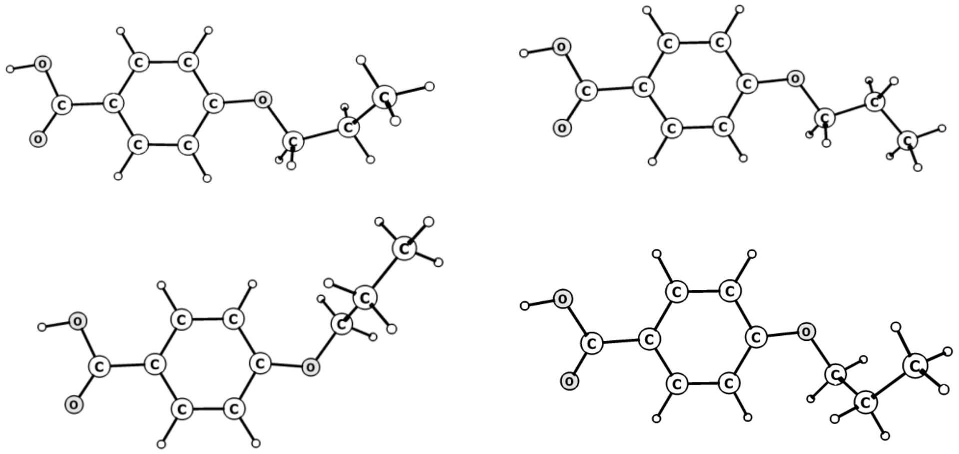

Figure S2. Conformers differing in the structure of the $–OC_3H_7$ substituent

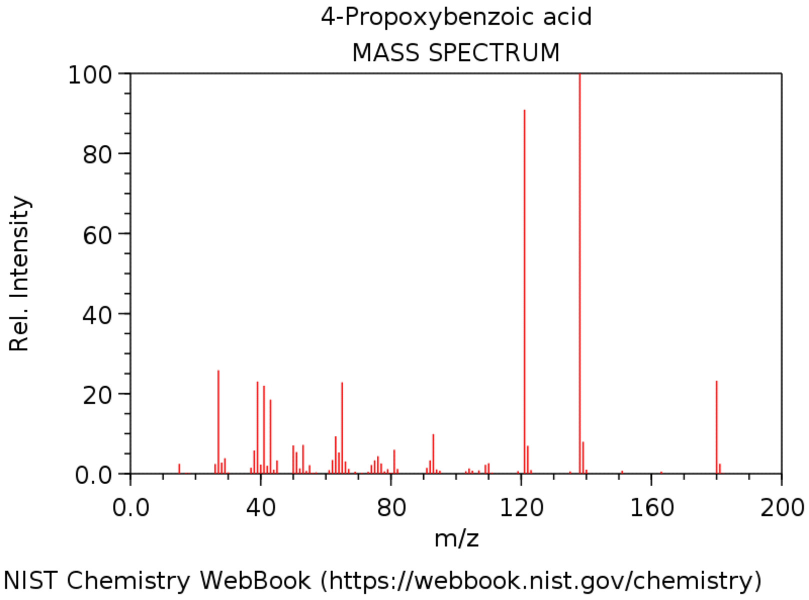

Figure S3. Mass spectrum of p-n-propyloxybenzoic acid (POBA) from the NIST database.

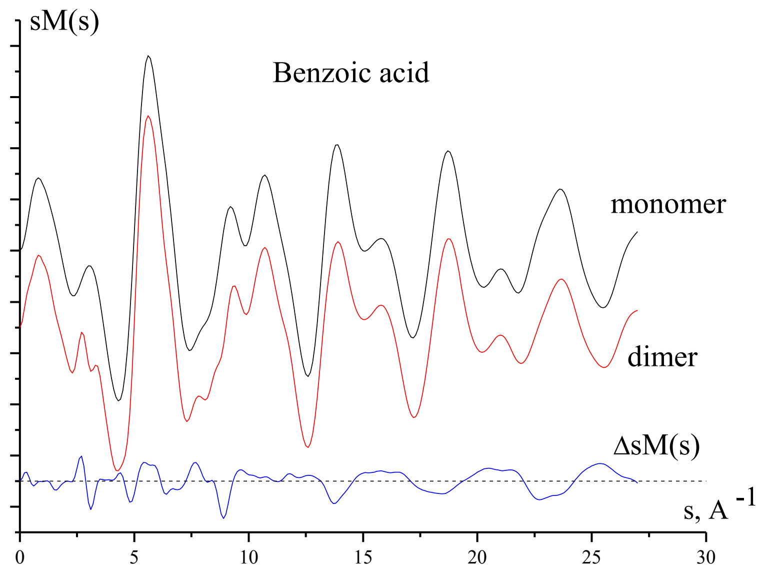

Figure S4. Theoretical curves of the molecular scattering intensity sM(s) of monomer, dimer of BA acid and their difference ΔsM(s).

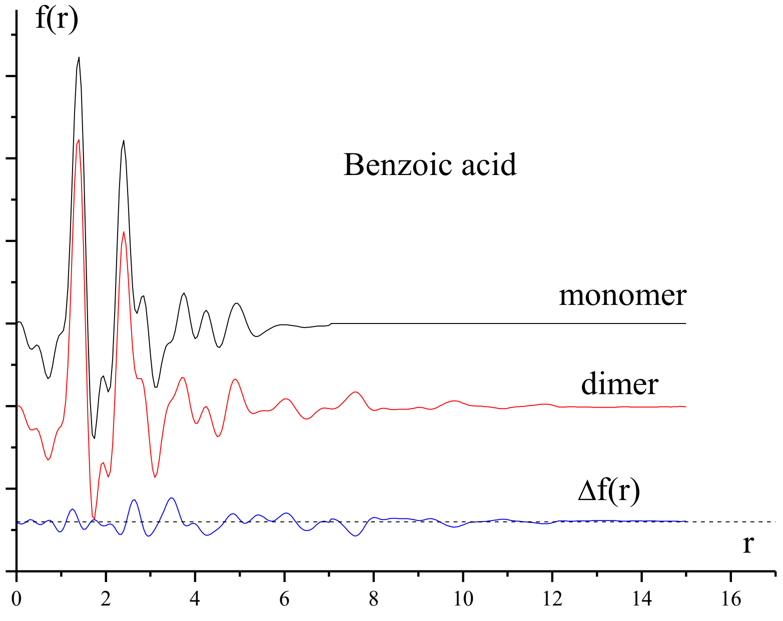

Figure S5. Theoretical curves of the radial distribution f(r) of monomer, dimer of BA acid and their difference Δf (r).

Table S1. Geometric characteristics of HBs and energy of dimerization of carboxylic acids according to the results of quantum chemical calculations

dimer	r(O-H) Å	r(H···O) Å	r(O···O) Å	O-H···O°	ΔE_{dim}[a] kcal/mol
AA···AA	1.008	1.665	2.673	178.9	-16.2
BA···BA	1.010	1.640	2.650	177.9	-17.0
POBA···POBA	1.010	1.648	2.647	177.6	-17.0

[a] $\Delta E_{dim} = E_{dim} - 2E_{mon}$; E_{dim} - the optimized energy of dimer, E_{mon} - the optimized energy of monomer
The interaction energy ΔE_{IMI} in POBA dimer, calculated taking into account BSSE, is -20.3 kcal/mol

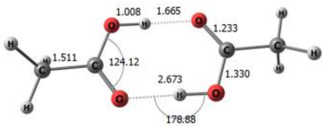

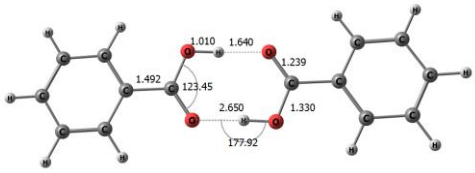

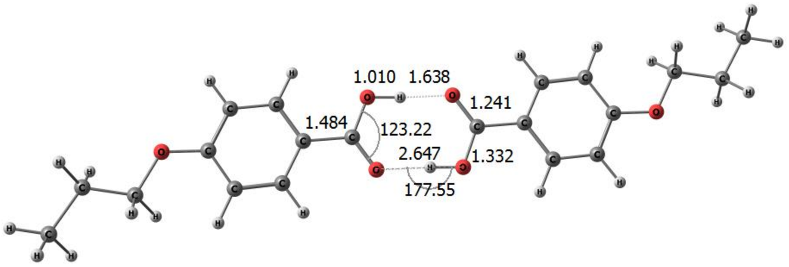

Table S2. Energy of donor-acceptor interaction of orbitals $E^{(2)}$ and energy of steric repulsion E_{steric} in POBA acid dimer (in kJ/mol)

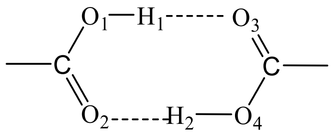

Interacting orbitals	Energy of donor-acceptor interaction of orbitals $E^{(2)}$	Interacting orbitals	Energy of steric repulsion E_{steric}
LP_2(O3) + σ*(O1-H1)	97.1	LP_2(O3) + σ(O1-H1)	55.3
LP_1(O3) + σ*(O1-H1)	33.9	LP_1(O3) + σ(O1-H1)	21.8
		LP_2(O2) + LP_2(O3)	4.2/2
$\Sigma E^{(2)}$ per one hydrogen bond	131.0	ΣE_{steric} per one hydrogen bond	79.2
$E_{HB} = \Sigma E^{(2)} - \Sigma E_{steric}$		51.8	

Note S1. The energies of intermolecular interaction in different aromatic carboxylic acids

In [29] for cyclic dimers of p-n-propyloxycinnamic, p-n-propyloxybenzoic and p-n-propylbenzoic acids we calculated the energies of intermolecular interaction (93.7, 92.9, 92.5 kJ mol^{-1}, CAM-B3LYP/6-311++G**) and parameters of H-bonds (r(H···O)= 1.630, 1.631, 1.633 Å). The HB parameters for three dimers are the same and do not depend on the nature of their core. In the same work, we show the influence of the calculation level (method/basis B3LYP, CAM-B3LYP, B97D/6-311++G**, cc-pVTZ) on the energies of intermolecular interaction (85.8–98.3 kJ mol–1).

The calculated value of 89 kJ/mol (B97D/6-311++G**) corresponds to the averaged values of all tests.

Note S2. Details of VibModule program modification for calculation of the vibrational parameters of supermolecules containing intermolecular hydrogen bonds.

Quote by [29]. "The modified version of the VibModule program [18] allows to solve vibrational task for molecules containing fragments with interaction similar to intermolecular (as in the case of the considered H-complexes). In old version of VibModule for supramolecules it is possible that some pairs of bound atoms (for example O···H) are absent in the list of bonds stretching coordinates, leading to the generation of an incomplete set of other types of internal coordinates. To solve this problem, an additional option has been introduced in VibModule program that allows to specify the pairs of atoms that need to be added to the list of internal the bond stretching coordinates. The generation of internal coordinates began with the determination of pairs of bound atoms. For this, all possible internuclear distances are calculated from Cartesian coordinates, and if the given internuclear distance is less than or equal to the sum of the van der Waals radii of the considered pair of atoms, this pair is added to the list of the bond stretching coordinates. The generation of the remaining types of internal coordinates (bending, out-of-plane bend, torsion, linear chain bending) is carried out using a refined set of pairs of bound atoms."
